# Supplementary material for: CO2-triggered reversible transformation of soft elastomers into rigid and highly fluorescent plastics
Source: Nat Commun. 2025 Nov 11;16:9582. doi: 10.1038/s41467-025-65495-4 (PMC12606198; doi:10.1038/s41467-025-65495-4)
Supplement: Supplementary file 1 — Supplementary Information [file 41467_2025_65495_MOESM1_ESM.pdf]

Supplementary Information for

CO_2-triggered reversible transformation of soft elastomers into rigid and highly fluorescent plastics

Yohei Miwa[1,*], Kazuma Okada[2], Takumi Hayashi[3], Kei Hashimoto[1], Hikaru Okubo[4], Hiroshi Takase[5], Katsuhiro Yamamoto[6,7], Ken Nakano[4], and Shoichi Kutsumizu[1]

[1]*Department of Chemistry and Biomolecular Science, Faculty of Engineering, Gifu University, Yanagido, Gifu 501-1193, Japan.*

[2]*Department of Engineering, Graduate School of Engineering, Gifu University, Yanagido, Gifu 501-1193, Japan.*

[3]*Department of Materials Science and Processing, Graduate School of Natural Science and Technology, Gifu University, Yanagido, Gifu 501-1193, Japan.*

[4]*Faculty of Environment and Information Sciences, Yokohama National University, Yokohama240-8501, Japan.*

[5]*Graduate School of Medical Sciences Core Laboratory, Nagoya City University, Kawakami, Mizuho-cho, Mizuho-ku, Nagoya, 467-0001, Japan.*

[6]*Department of Life Science and Applied Chemistry, Graduated School of Engineering, Nagoya Institute of Technology, Gokiso-cho, Showa-ku, Nagoya 466-8555, Japan*

[7]*Present address: Department of Materials Chemistry, Faculty of Engineering, Shinshu University, 4-17-1 Wakasato, Nagano, Nagano 380-8553, Japan.*

*Author to whom correspondence should be addressed.

E-mail: miwa.yohei.y6@f.gifu-u.ac.jp

Table of Contents

Supplementary Figure 1. GPC traces of PDMS–L, PDMS–M, and PDMS–H.

Supplementary Figure 2. Photographs of H(*n*), M(*n*) and L(*n*) samples.

Supplementary Figure 3. Experimental SAXS profiles of M(*n*) and b, L(*n*) samples with and without CO_2 exposure.

Supplementary Figure 4. TEM image of M(50).

Supplementary Figure 5. Difference FTIR spectra of H(40) during $N_2 \rightarrow CO_2$ process.

Supplementary Figure 6. Two-dimensional infrared correlation spectra recorded during $CO_2 \rightarrow N_2$ process.

Supplementary Figure 7. Change in the weight of bulk-state PEI during the $N_2 \rightarrow CO_2$ process, used to monitor CO_2 uptake.

Supplementary Figure 8. Weight changes of M(*n*) and L(*n*) samples during the $N_2 \rightarrow CO_2$ process at 25 °C.

Supplementary Figure 9. Change in the weight of CO_2-cured H(40) during heating at 5 °C min^{-1} under N_2 flow.

Supplementary Figure 10. Weight change of L(70) during the $N_2 \rightarrow CO_2$ process at certain temperatures. CO_2 capture capacity of L(70) measured at 25 °C, 60 °C, and 90 °C.

Supplementary Figure 11. CO_2-capture performance of H(30) with 10 cycles of CO_2 adsorption and release by heating.

Supplementary Figure 12. Change in the shear storage modulus (*G*′) of PEI at 25 °C during the $N_2 \rightarrow CO_2$ process.

Supplementary Figure 13. MDSC thermograms of PDMS-H with and without CO_2.

Supplementary Figure 14. Change in the tensile storage modulus (*E*') of H(24) at 25 °C and 1 Hz during the Air → CO_2 process. The humidity of the air is 50%.

Supplementary Figure 15. Stress–strain curves of CO_2-cured H(30) recorded immediately after curing (solid line) and after one month (thick dashed line) and two months (thin dashed line) of storage in air. The Young's modulus (*E*) of CO_2-cured H(30) gradually decreases with storage time.

Supplementary Figure 16. Change in the tensile storage modulus (*E*') of H(24) at 25 °C and 1 Hz during the N_2 → CO_2 and CO_2 → N_2 processes. Following CO_2 exposure, the sample was heated to 100 °C and cooled back to 25 °C under N_2 flow.

Supplementary Figure 17. Stress–strain curves of CO_2-cured a, M(*n*) and b, L(*n*) samples. c, Strain at break, Young's modulus (*E*), and toughness of L(*n*), M(*n*), and H(*n*) samples as functions of PEI content.

Supplementary Figure 18. Selected adhesion-vs.-time curves for H(40) at 25 °C in different gases.

Supplementary Figure 19. ^{1}H-NMR spectra of PDMS–L, PDMS–M, and PDMS–H.

Supplementary Figure 20. ^{13}C-NMR spectrum of PEI.

Supplementary Figure 21. FTIR spectra of PEI, H(*n*) samples, and PDMS–H.

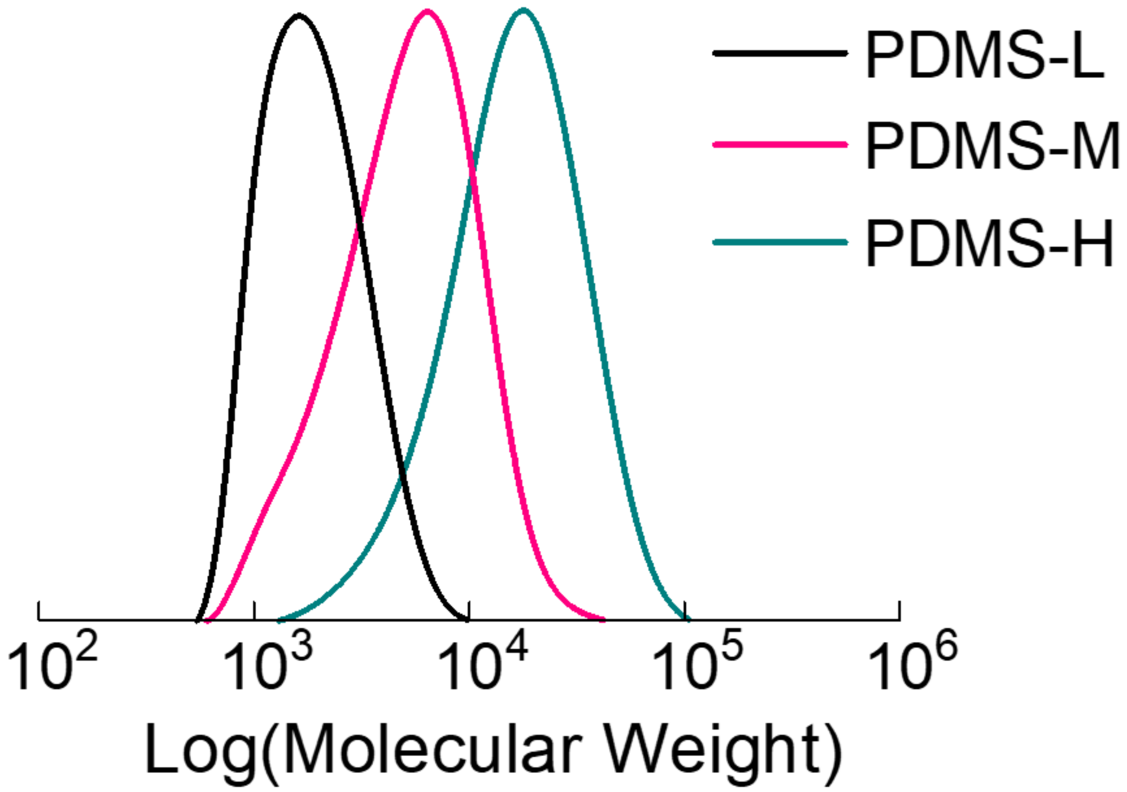

Supplementary Figure 1 | Molecular weight measurement for telechelic epoxy PDMS. GPC traces of PDMS–L, PDMS–M, and PDMS–H.

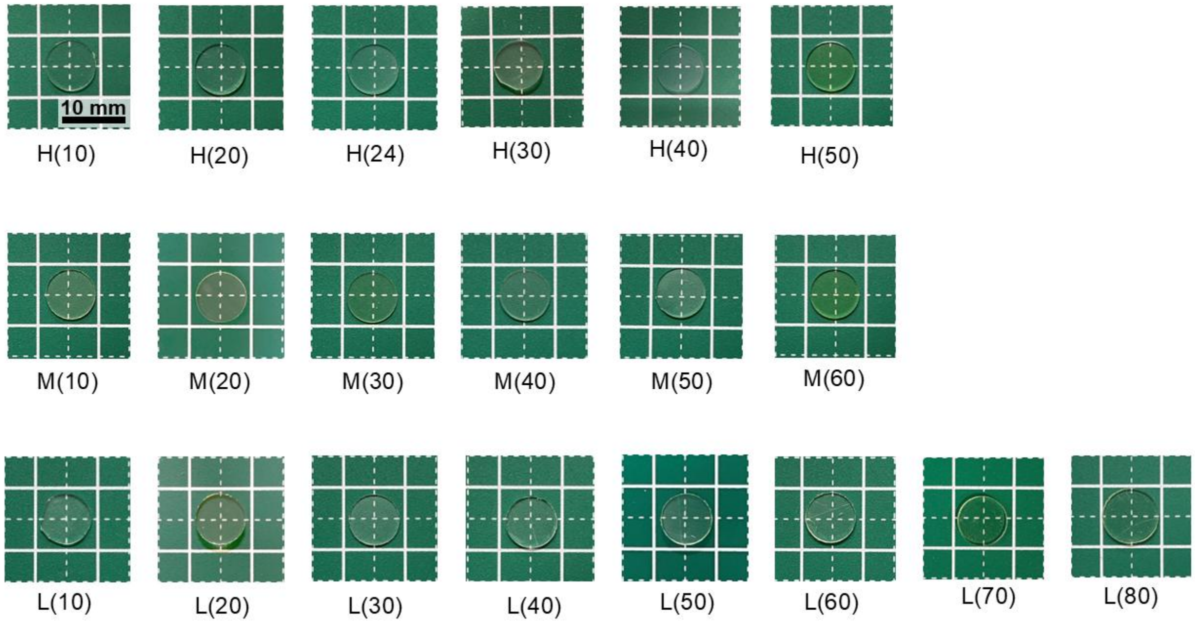

Supplementary Figure 2 | Appearance of prepared samples. Photographs of H(*n*), M(*n*) and L(*n*) samples

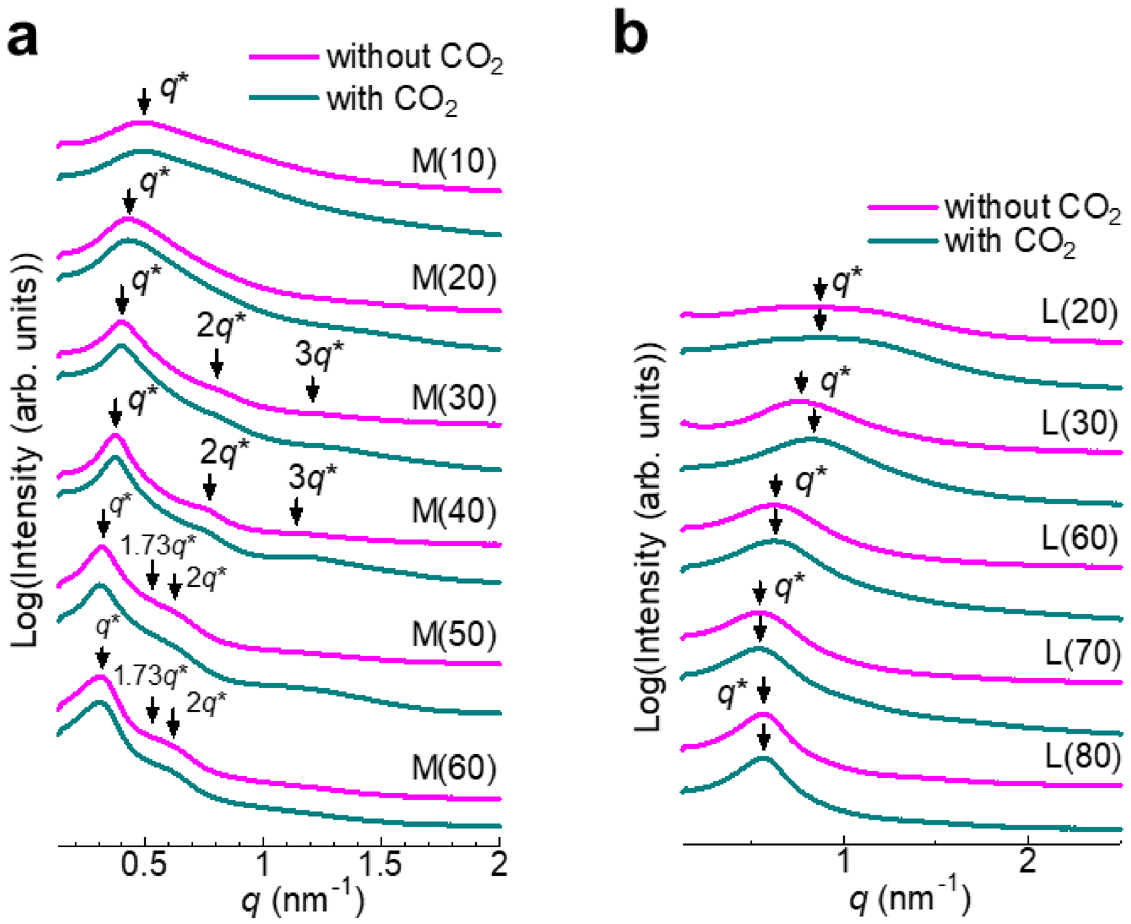

Supplementary Figure 3 | SAXS profiles of M(*n*) and L(*n*) samples. Experimental SAXS profiles of **a,** M(*n*) and **b,** L(*n*) samples with and without CO_2 exposure.

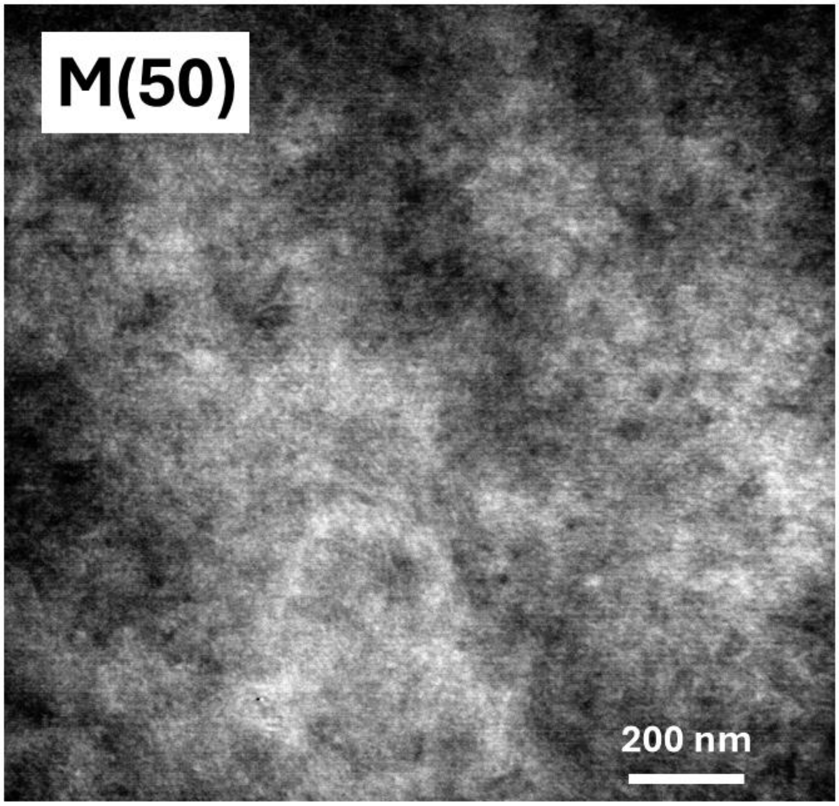

Supplementary Figure 4 | Morphology of M(50). TEM image of M(50).

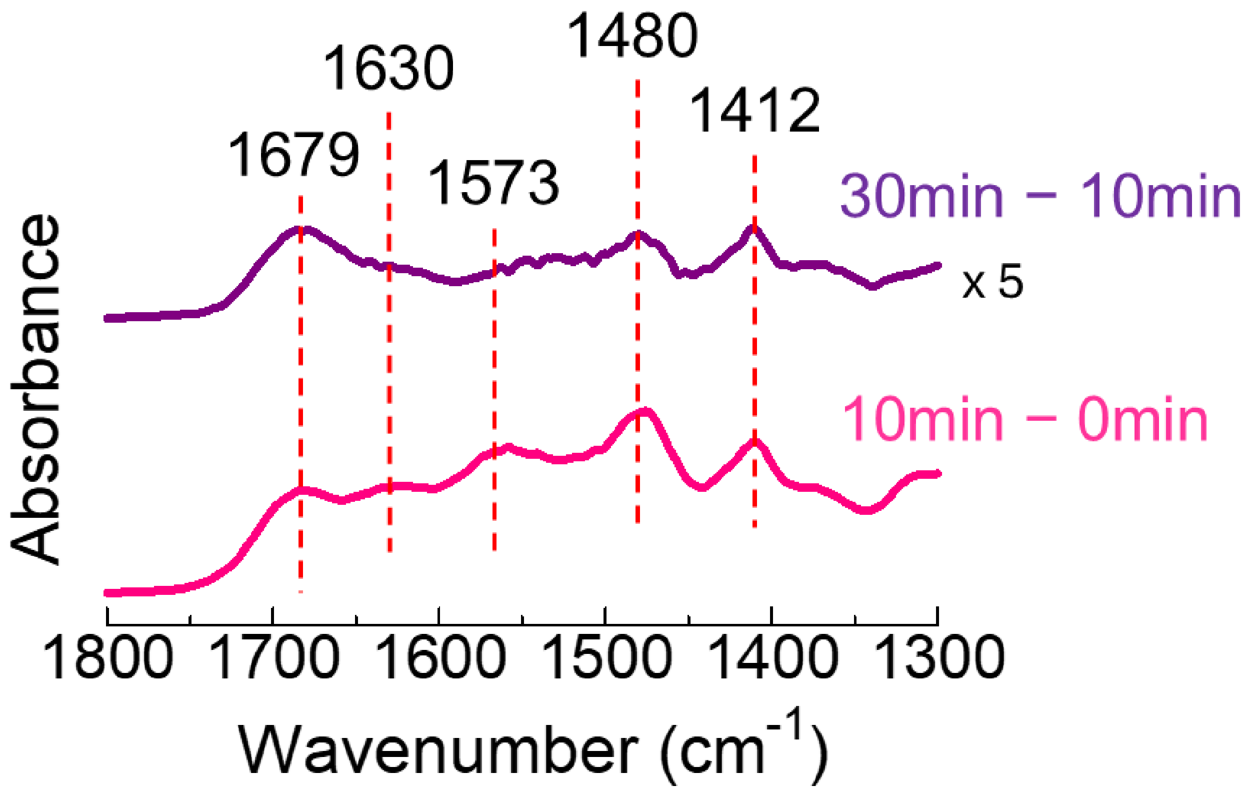

Supplementary Figure 5 | Formations of ammonium carbamates and carbamic acids in H(40) upon CO_2 exposure. Difference FTIR spectra of H(40) during $N_2 \rightarrow CO_2$ process.

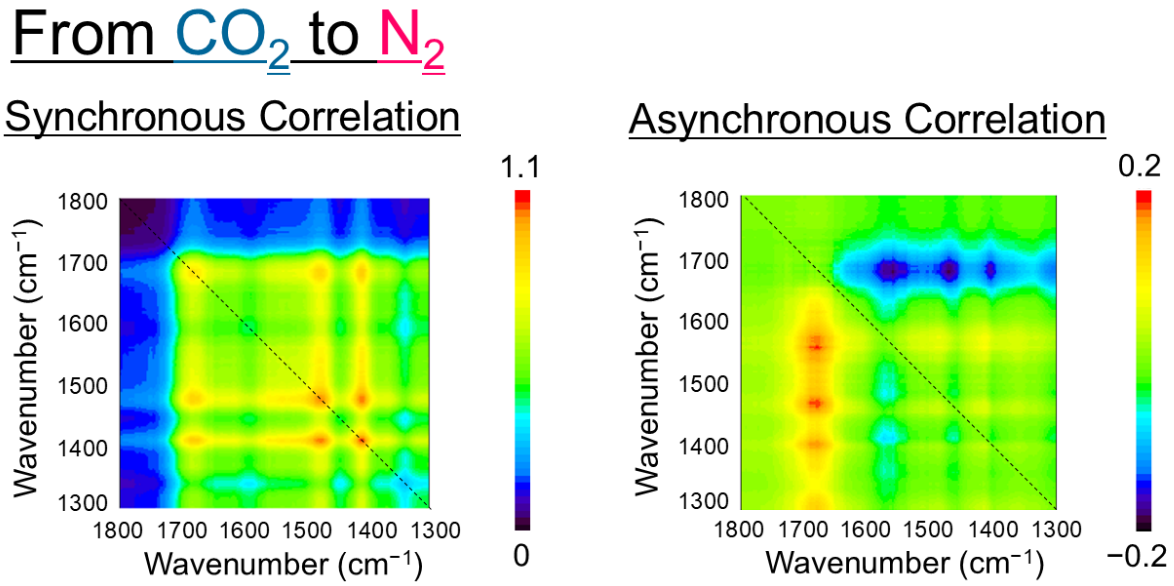

Supplementary Figure 6 | Deformations of ammonium carbamates and carbamic acids in H(40) under N_2 flow. Two-dimensional infrared correlation spectra recorded during $CO_2 \rightarrow N_2$ process.

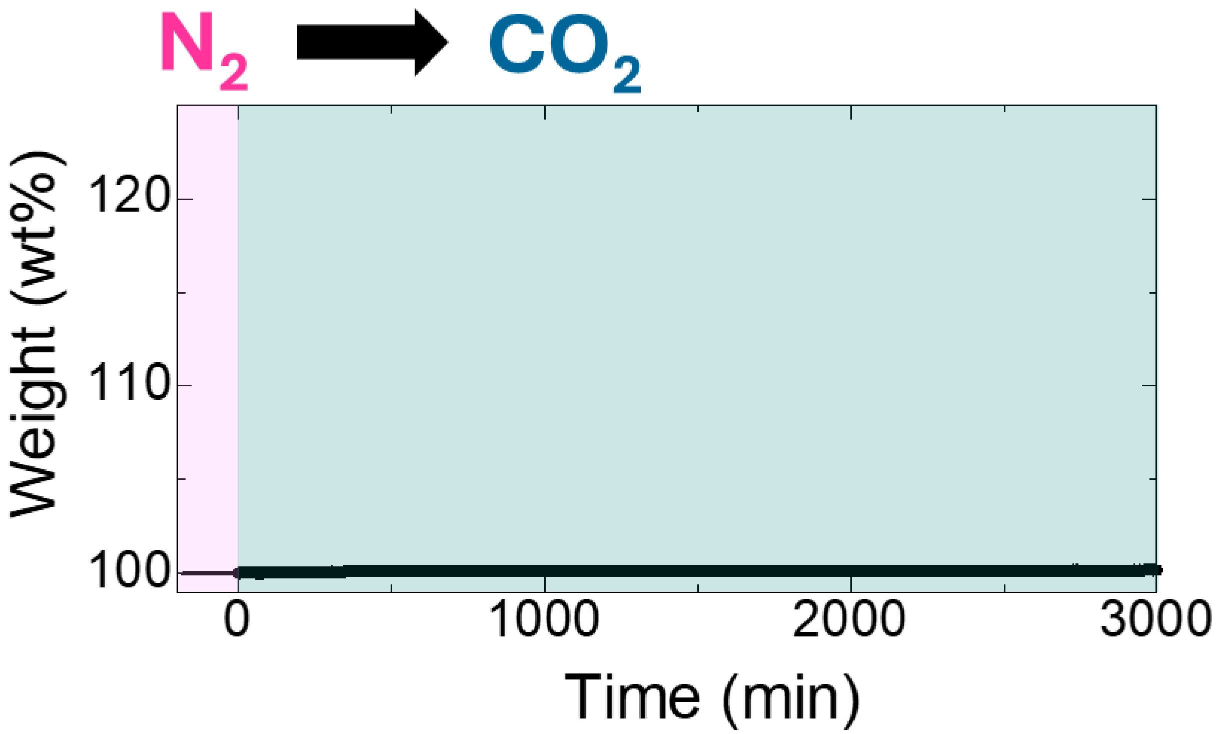

Supplementary Figure 7 | CO_2-capture behavior of bulk PEI ($M_n \approx$ 10,000). Change in the weight of bulk-state PEI during the $N_2 \rightarrow CO_2$ process, used to monitor CO_2 uptake.

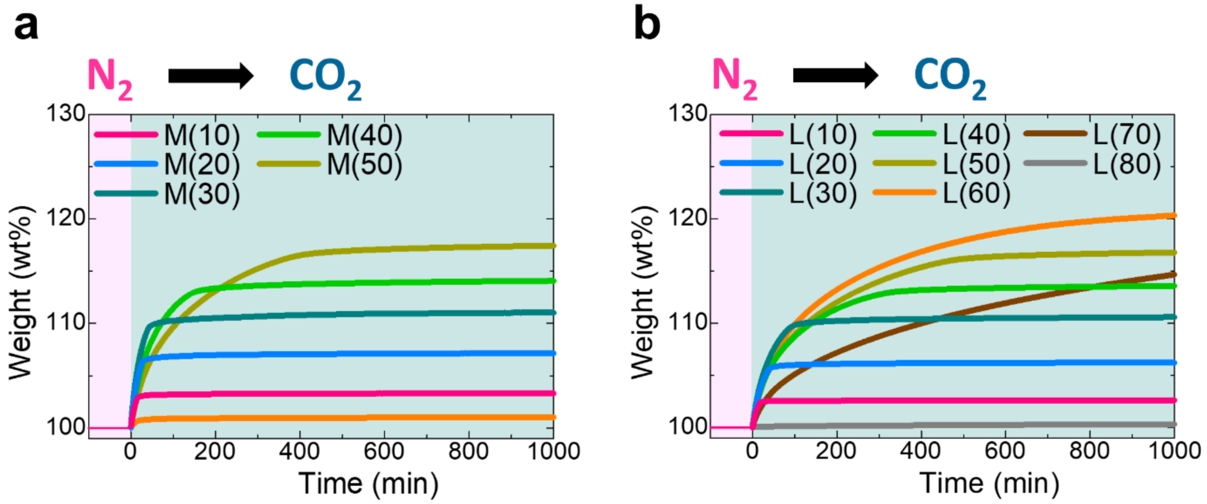

Supplementary Figure 8 | CO2-capture performances of M(*n*) and L(*n*) samples. Weight changes of a, M(*n*) and b, L(*n*) samples during the $N_2 \rightarrow CO_2$ process at 25 °C.

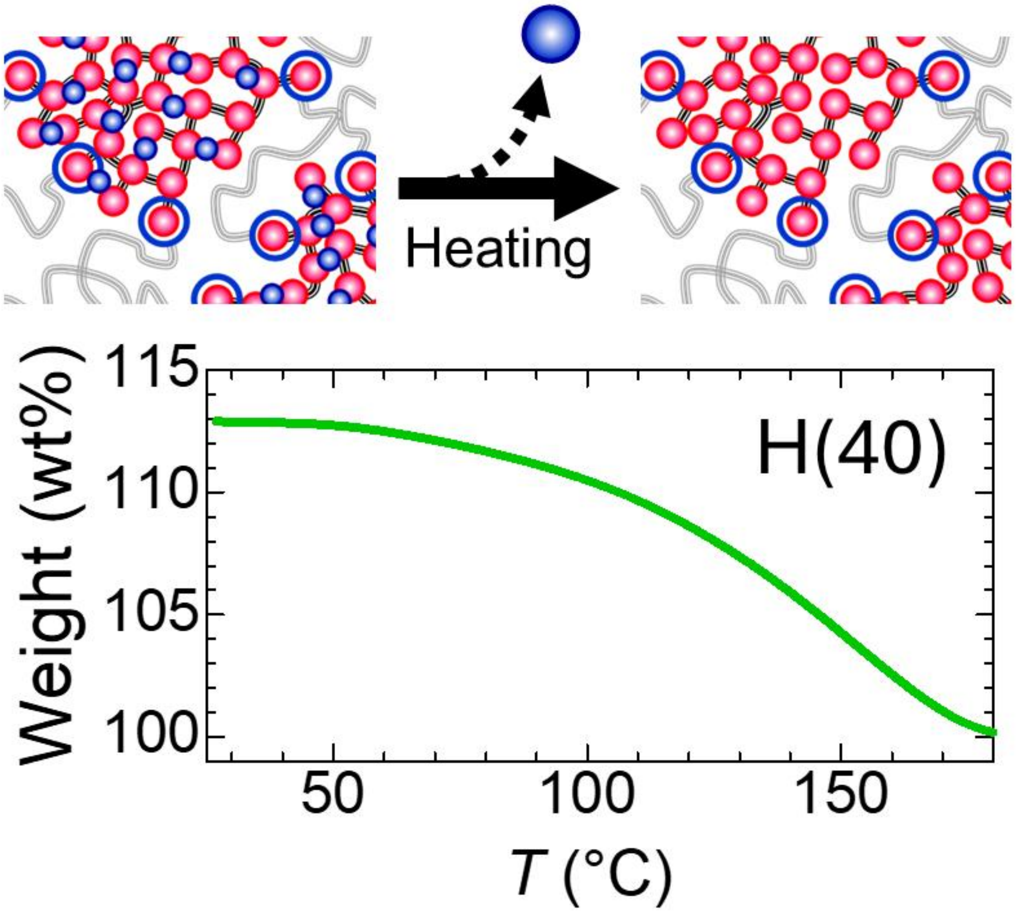

Supplementary Figure 9 | Thermal analysis of CO_2-cured H(40). Change in the weight of CO_2-cured H(40) during heating at 5 °C min^{-1} under N_2 flow.

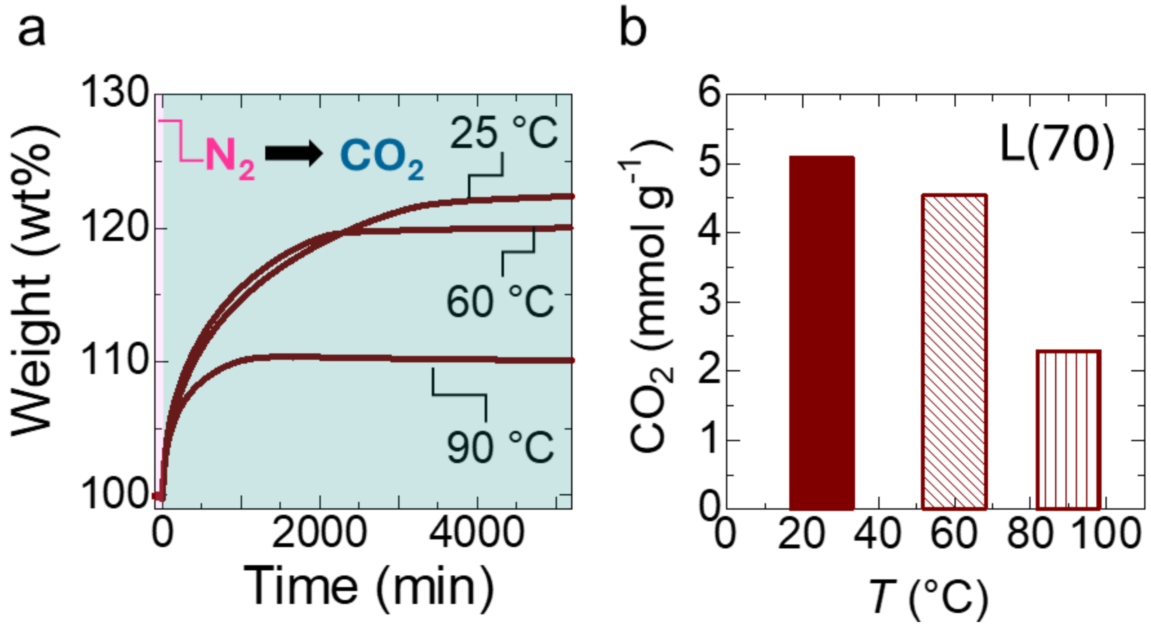

Supplementary Figure 10 | Effect of temperature on the CO_2-capture performance of L(70). a, Weight change of L(70) during the N_2 → CO_2 process at certain temperatures. **b,** CO_2 capture capacity of L(70) measured at 25 °C, 60 °C, and 90 °C.

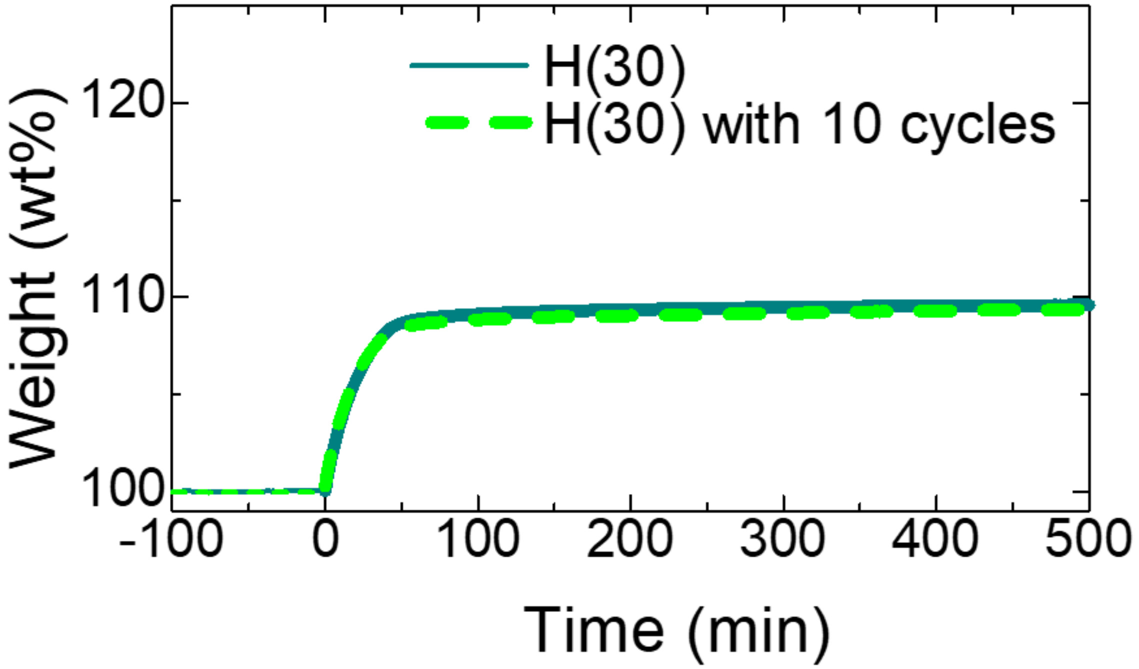

Supplementary Figure 11 | CO_2-capture performance of H(30) with 10 cycles of CO_2 absorption and release by heating. The H(30) sheet absorbed CO_2 for one hour and heated at 150 °C for 30 min under N_2 flow in each cycle.

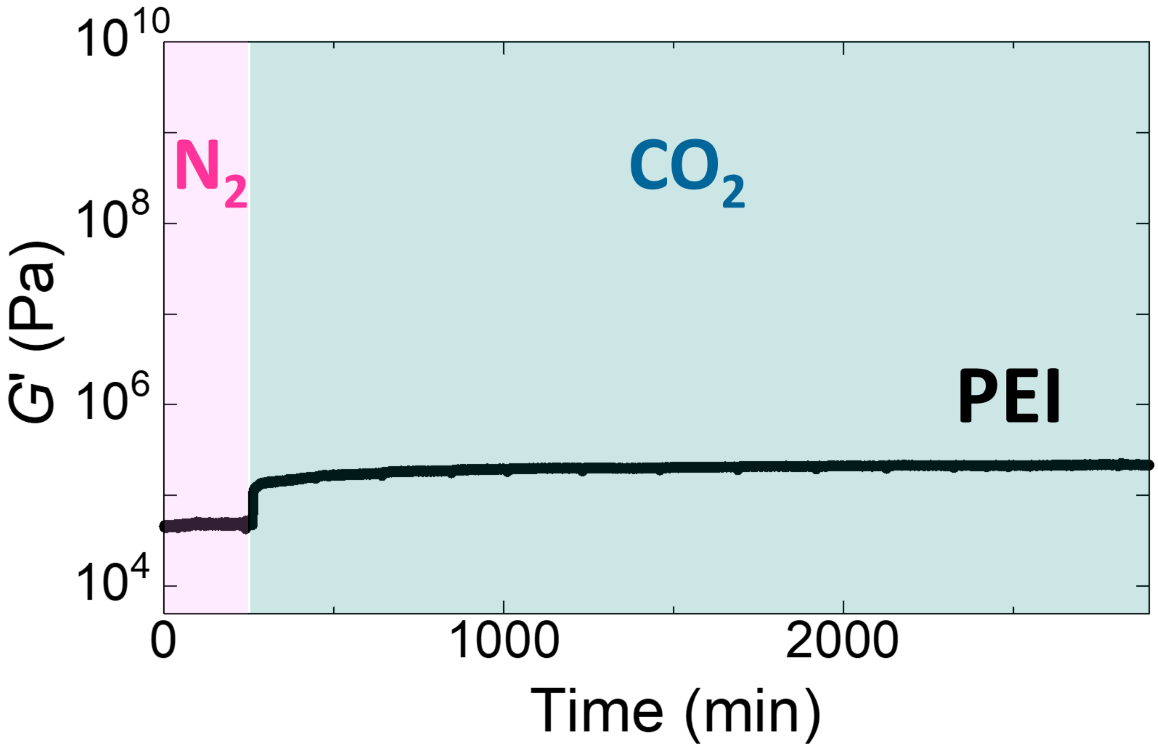

Supplementary Figure 12 | Effect of CO_2 exposure on the elastic modulus of PEI. Change in the shear storage modulus (G') of PEI at 25 °C during the $N_2 \rightarrow CO_2$ process.

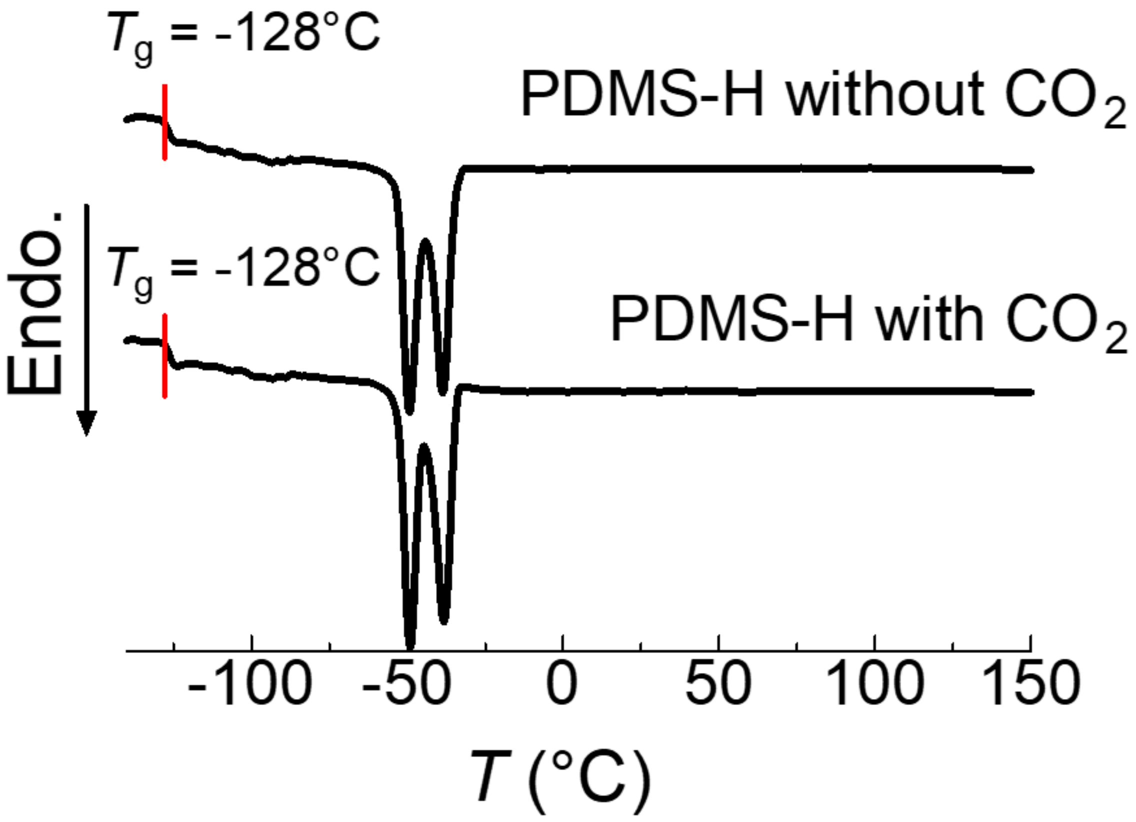

Supplementary Figure 13 | Effect of CO_2 on thermal property of PDMS. MDSC thermograms of PDMS-H with and without CO_2.

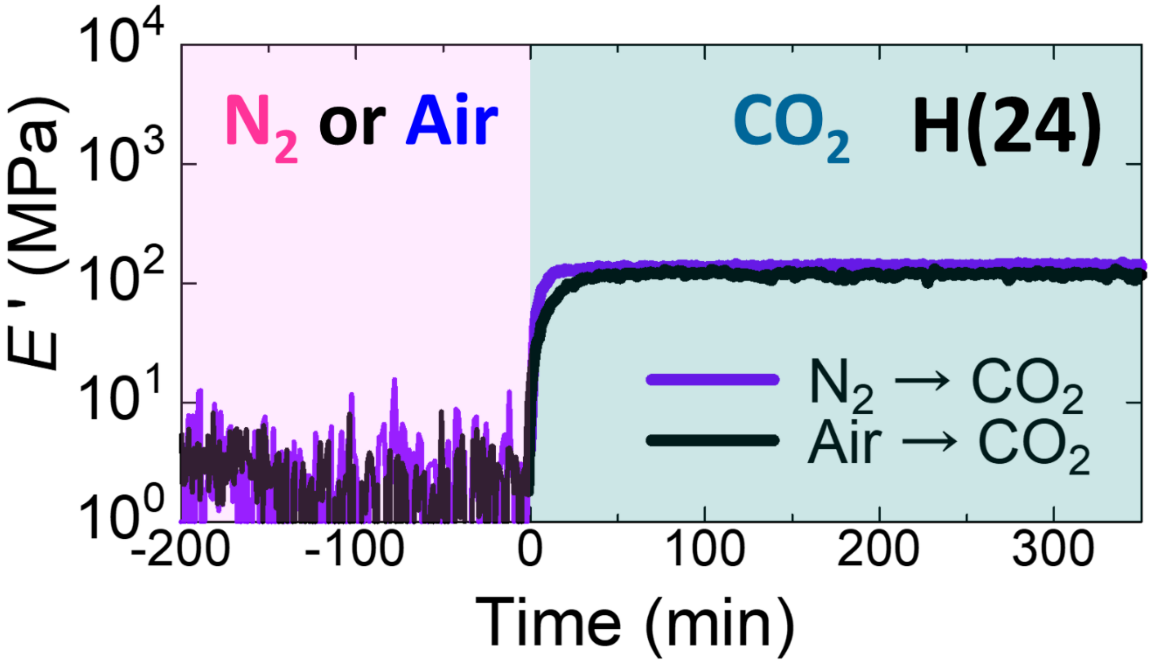

Supplementary Figure 14 | Effect of air on CO_2-curing behavior. Changes in the tensile storage modulus (*E*') of H(24) at 25 °C and 1 Hz during the Air → CO_2 and N_2 → CO_2 processes. The humidity of the air is 50%.

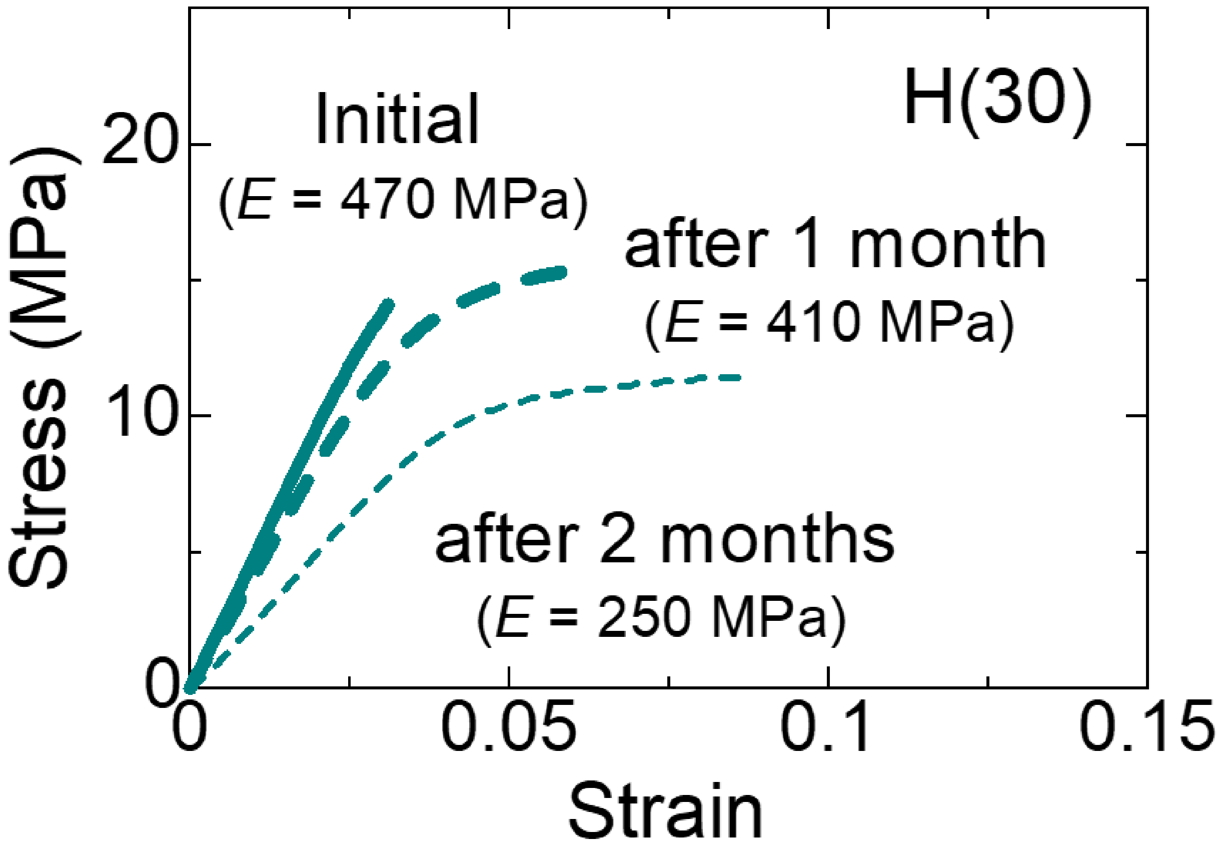

Supplementary Figure 15 | Influence of storage duration on the mechanical behavior of H(30). Stress–strain curves of CO_2-cured H(30) recorded immediately after curing (solid line) and after one month (thick dashed line) and two months (thin dashed line) of storage in air. The Young's modulus (*E*) of CO_2-cured H(30) gradually decreases with storage time.

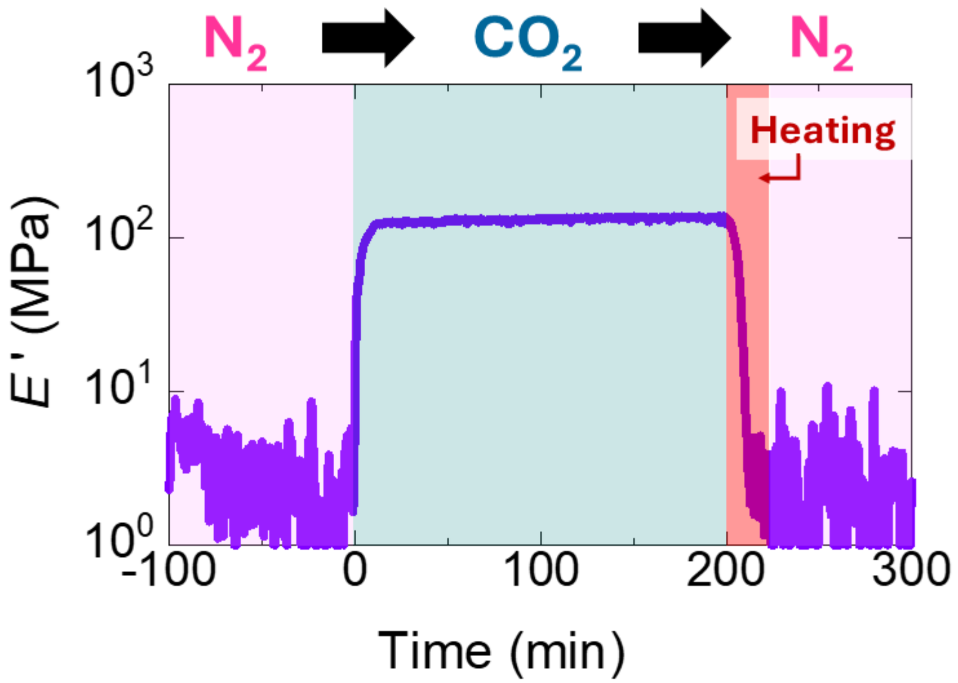

Supplementary Figure 16 | Mechanical cyclability of H(24). Change in the tensile storage modulus (E') of H(24) at 25 °C and 1 Hz during the $N_2 \rightarrow CO_2$ and $CO_2 \rightarrow N_2$ processes. Following CO_2 exposure, the sample was heated to 100 °C and cooled back to 25 °C under N_2 flow.

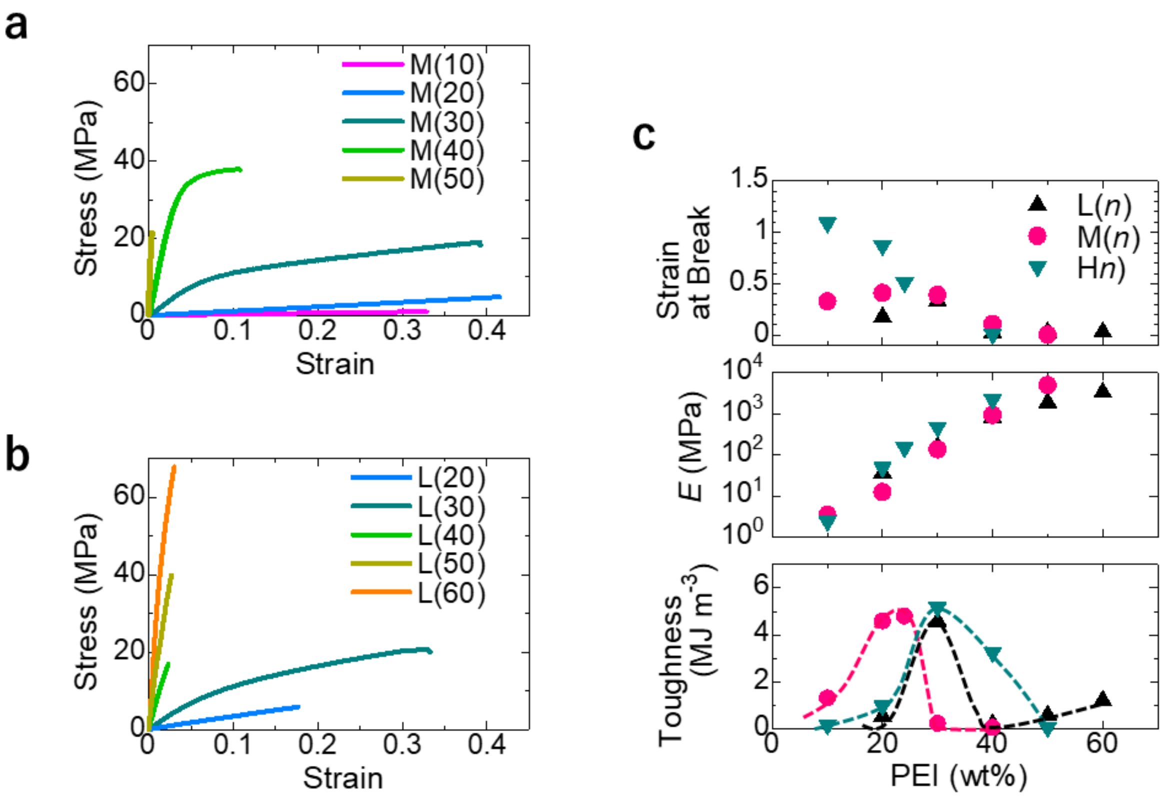

Supplementary Figure 17 | Tensile behavior of CO_2-cured polymer materials with short PDMS chains. Stress–strain curves of CO_2-cured **a,** M(*n*) and **b,** L(*n*) samples. **c,** Strain at break, Young's modulus (*E*), and toughness of L(*n*), M(*n*), and H(*n*) samples as functions of PEI content.

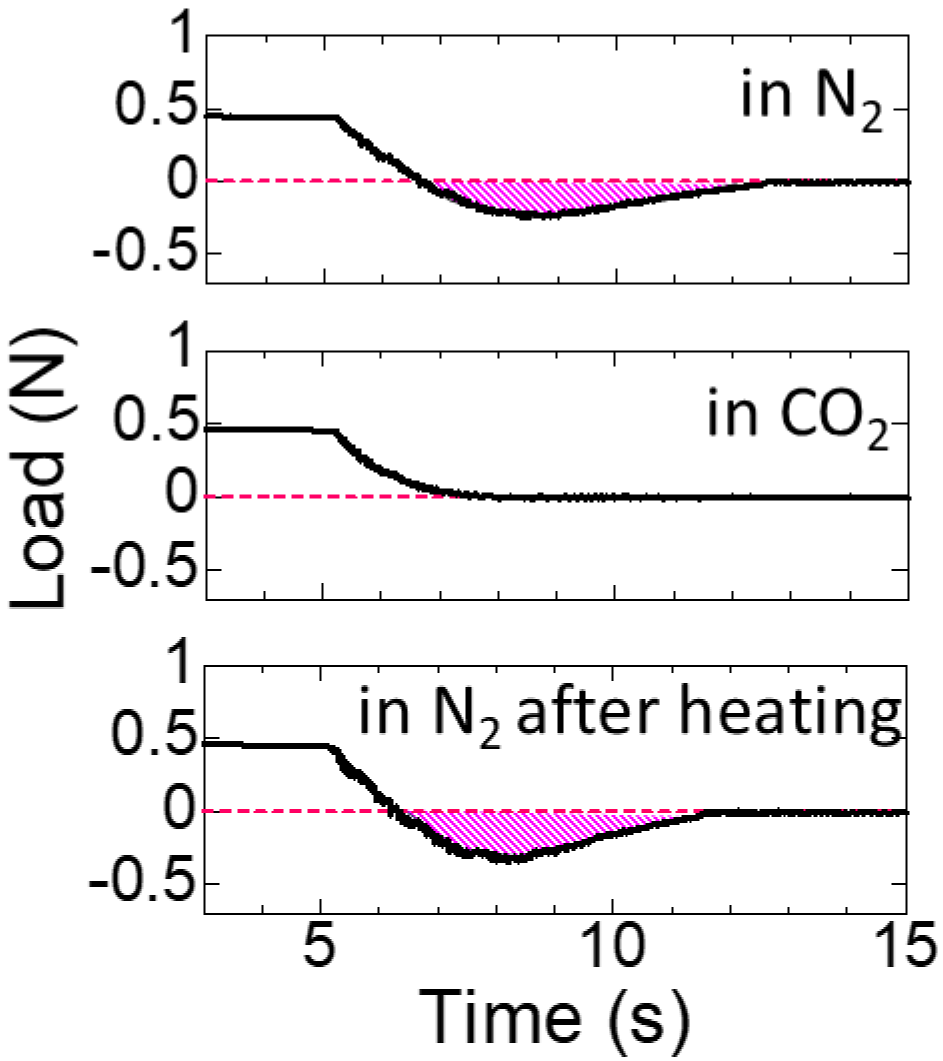

Supplementary Figure 18 | Gas switchable adhesion behavior of H(40). Selected load-vs.-time curves for H(40) at 25 °C with different gases.

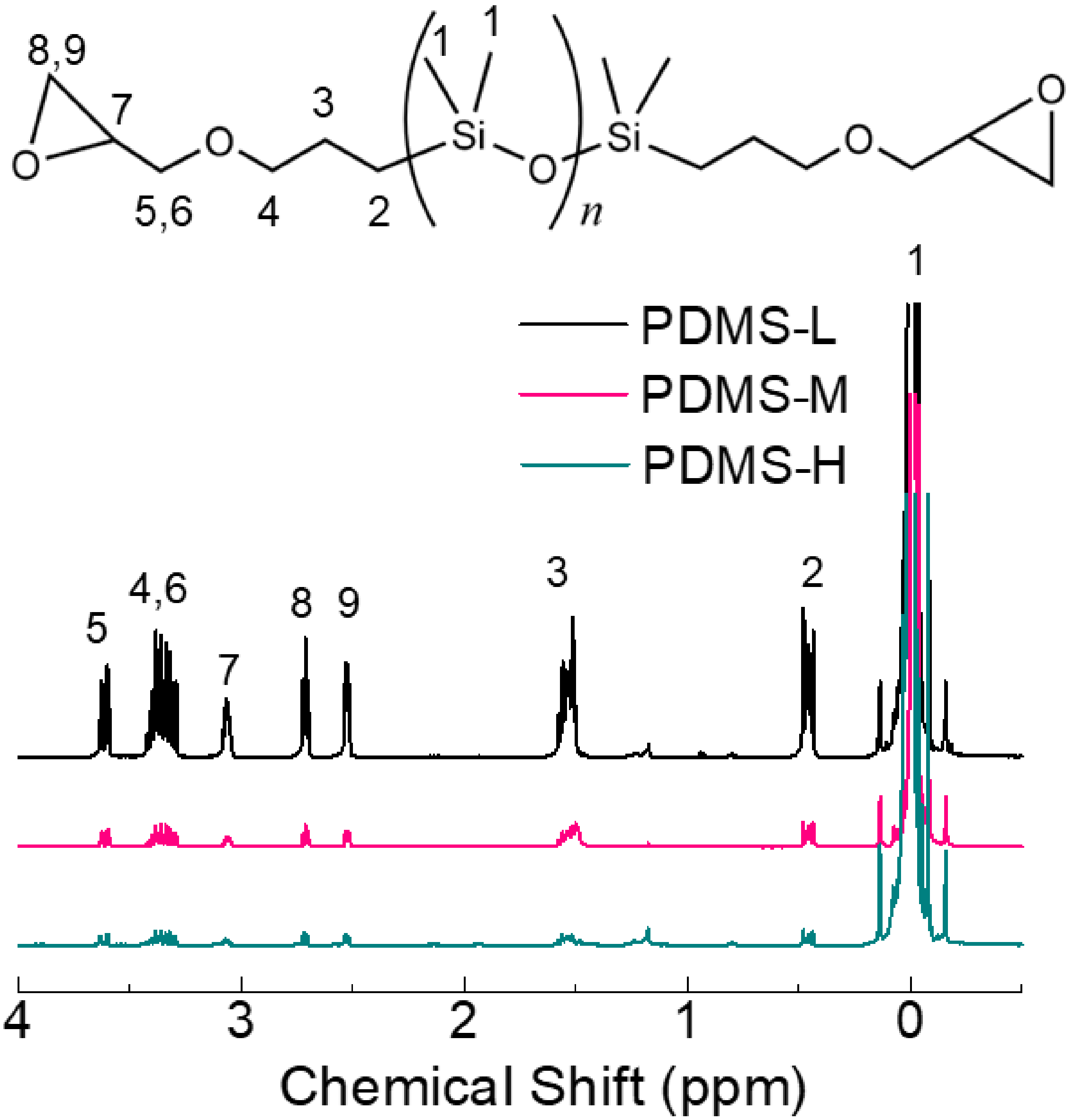

Supplementary Figure 19 | Structural analysis of telechelic epoxy-PDMS. ^{1}H-NMR spectra of PDMS–L, PDMS–M, and PDMS–H. A 400 MHz JEOL-ECS400 spectrometer was used with $CDCl_3$ containing tetramethylsilane.

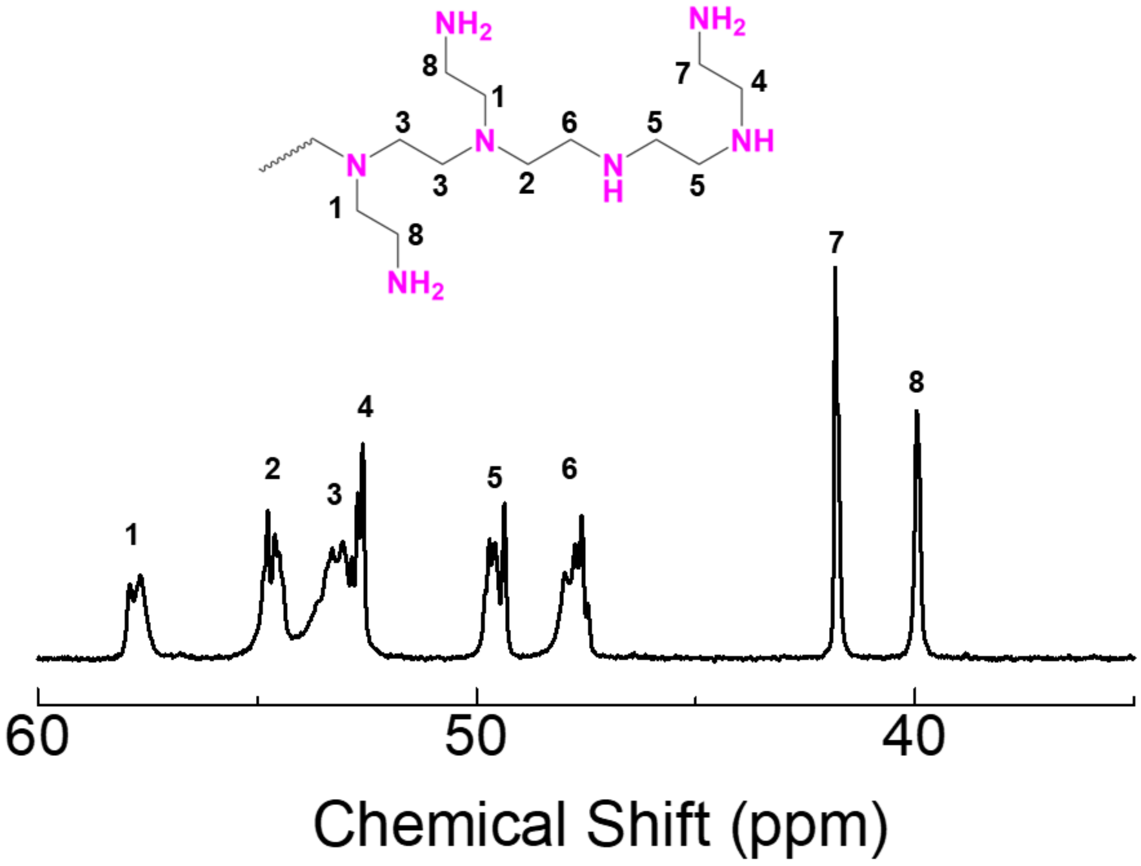

Supplementary Figure 20 | Microstructure analysis of PEI. ^{13}C-NMR spectrum of PEI. A 400 MHz JEOL-ECS400 spectrometer was used with $CDCl_3$ containing tetramethylsilane.

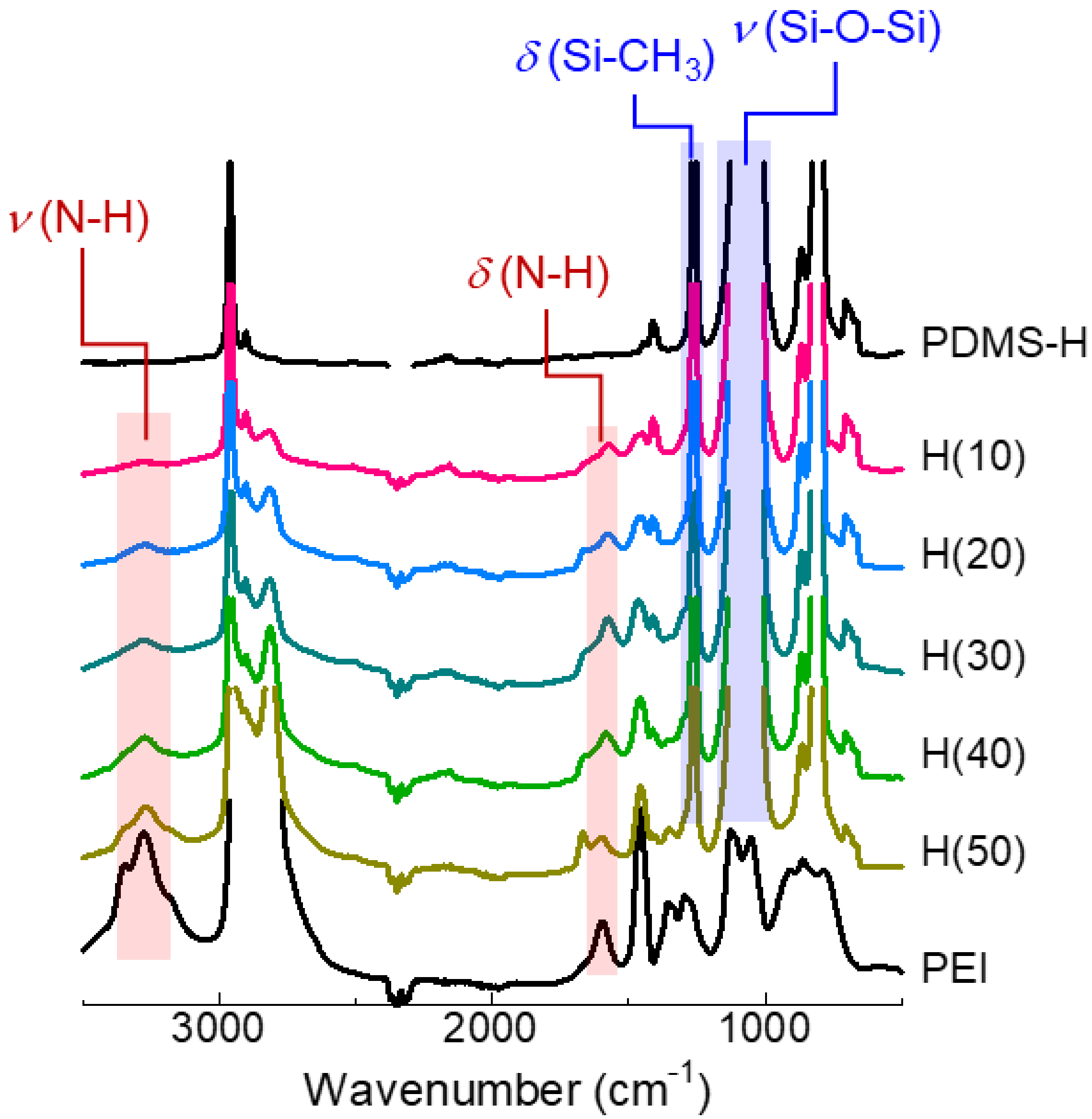

Supplementary Figure 21 | FTIR analysis of synthesized materials. FTIR spectra of PEI, H(*n*) samples, and PDMS–H.
