# Supplementary material for: CO2-triggered reversible transformation of soft elastomers into rigid and highly fluorescent plastics
Source: Nat Commun. 2025 Nov 11;16:9582. doi: 10.1038/s41467-025-65495-4 (PMC12606198; doi:10.1038/s41467-025-65495-4)
Supplement: Supplementary file 2 — Description of Additional Supplementary Files [file 41467_2025_65495_MOESM2_ESM.pdf]

Description of Additional Supplementary Files

Supplementary Movie 1. Demonstration of a 2 kg dumbbell being lifted by an H(30) sheet without CO_2 exposure.

Supplementary Movie 2. Demonstration of a 2 kg dumbbell being lifted by an H(30) sheet with CO_2 exposure.

Supplementary Movie 3. Effect of CO_2 on surface property of H(40) sheet. The stickiness of the H(40) surface can be instantly removed by blowing CO_2.
